# Increased Serum Uric Acid over five years is a Risk Factor for Developing Fatty Liver

**DOI:** 10.1038/s41598-018-30267-2

**Published:** 2018-08-06

**Authors:** Thomas Jensen, Koichiro Niwa, Ichiro Hisatome, Mehmet Kanbay, Ana Andres-Hernando, Carlos A. Roncal-Jimenez, Yuka Sato, Gabriela Garcia, Minoru Ohno, Miguel A. Lanaspa, Richard J. Johnson, Masanari Kuwabara

**Affiliations:** 1 Division of Renal Diseases and Hypertension, School of Medicine, University of Colorado Denver, Aurora, CO USA; 2 Division of Endocrinology, Diabetes, and Metabolism, School of Medicine, University of Colorado Denver, Aurora, CO USA; 3 Cardiovascular Center, St. Luke’s International Hospital, Tokyo, Japan; 4 Division of Regenerative Medicine and Therapeutics, Tottori University Graduate School of Medical Sciences, Tottori, Japan; 5 Division of Nephrology, Department of Internal Medicine, Koç University School of Medicine, Istanbul, Turkey; 6 Department of Cardiology, Toranomon Hospital, Tokyo, Japan

## Abstract

The prevalence of fatty liver disease (FLD) is increasing. To clarify risk factors for developing FLD, we analyzed a database from healthy Japanese adults who had annual medical check-ups in 2004 and reexamined in 2009. We used the fatty liver index (FLI) to classify participants as FLD (FLI ≥60), borderline FLD (30≤ FLI <60), and normal liver (FLI <30). Subjects with hepatitis B or C virus infection and subjects with FLD at the baseline were excluded. The cumulative incidence of FLD from normal liver and from borderline FLD over five years were 0.65% (52/8,025) and 12.9% (244/1,888), respectively. After multiple adjustments, higher serum uric acid (SUA) (OR:1.92; 95% CI:1.40–2.63) and increased SUA change (OR:3.734; 95% CI:2.57–5.42) became risk factors for developing FLD from normal liver, as well as younger age and higher body mass index. The risk factors for developing FLD from borderline FLD were similar. Not only higher baseline SUA but also increased SUA change became independent risks for developing FLD.

## Introduction

Fatty liver disease (FLD) is emerging now as the number one cause of chronic liver disease especially in developed countries^1^. Patients with FLD have been found to have increased morbidity and mortality compared to non-FLD patients. Of note, patients with FLD have a 2–6 fold risk of fatal and nonfatal CVD^2–5^, a 2–3 fold risk of developing diabetes^6^, and an overall increased mortality of 57%^7^. FLD will soon eclipse Hepatitis C as the number one indication for liver transplant^8^.

Early identification of those at risk for FLD is necessary in aiding timely discussions about the disease, screening, and prevention especially through life style modifications. Traditional risk factors such as central obesity, insulin resistance, western diet (high fructose, high fat, high salt), sedentary lifestyle, and genetics are well established as having roles in the development and progression of FLD^9^. Yet despite the recent expansion into our understanding of FLD, the pathological development of FLD has not been fully explained^10^. Besides traditional risk factors such as weight and diabetes, it is well known that serum uric acid (SUA) has also been found to associate with FLD^11^. Many studies have shown that SUA is a risk factor for developing FLD^12–14^. However, it is unclear how SUA change over time affects the risk for developing FLD. This study checked our hypothesis that not only a higher SUA at baseline, but also increase in SUA over time are risk factors for developing FLD.

## Results

### Demographics of this study subjects

Table 1 shows the demographics with normal liver and borderline FLD at the baseline (in 2004). Compared to men with normal liver group, borderline FLD group are older, higher male gender, higher body mass index (BMI), higher rate of smoking and drinking habits, and have a higher prevalence of hypertension, diabetes mellitus, dyslipidemia, and chronic kidney disease, along with higher liver enzyme levels, and higher SUA levels.

**Table 1:** Demographics of study subjects.

Number of subjects	Total	Normal liver	Borderline fatty liver	p
	9,914	8,025	1,889	
Age	48.7 ± 10.6	48.1 ± 10.6	51.0 ± 10.4	<0.001
Male gender	41.6%	32.4%	80.6%	<0.001
Height (cm)	163.0 ± 8.4	161.9 ± 8.1	167.7 ± 8.0	<0.001
Weight (kg)	58.0 ± 10.2	55.3 ± 8.7	69.5 ± 7.9	<0.001
Body mass index (kg/m^2^)	21.7 ± 2.6	21.0 ± 2.3	24.7 ± 2.0	<0.001
Waist circumference (cm)	79.0 ± 7.9	77.0 ± 7.0	87.7 ± 5.1	<0.001
Smoking habits	34.4%	29.6%	54.9%	<0.001
Drinking habits	41.0%	37.6%	55.3%	<0.001
Hypertension	8.9%	6.7%	18.2%	<0.001
Diabetes mellitus	1.5%	0.9%	3.9%	<0.001
Dyslipidemia	30.5%	22.7%	63.9%	<0.001
Chronic kidney disease	2.0%	1.8%	3.1%	0.002
Fatty liver index	16.0 ± 15.3	9.63 ± 7.7	43.2 ± 8.5	<0.001
Triglyceride (mg/dL)	85.6 ± 47.4	72.6 ± 32.8	140.8 ± 59.1	<0.001
Total protein (g/dL)	7.13 ± 0.36	7.12 ± 0.36	7.21 ± 0.35	<0.001
Total bilirubin (mg/dL)	0.83 ± 0.30	0.82 ± 0.30	0.84 ± 0.29	0.054
Aspartate aminotransferase (IU/L)	21.5 ± 6.5	20.8 ± 6.1	24.5 ± 7.4	<0.001
Alanine transaminase (IU/L)	19.1 ± 10.9	16.9 ± 8.0	28.3 ± 15.5	<0.001
Gamma-glutamyl transferase (IU/L)	29.0 ± 26.5	22.9 ± 16.2	55.2 ± 41.6	<0.001
Alkaline phosphatase (IU/L)	190 ± 57	186 ± 56	208 ± 56	<0.001
Serum uric acid (mg/dL)	5.13 ± 1.33	4.87 ± 1.21	6.22 ± 1.24	<0.001
Serum uric acid change (mg/dL)	−0.025 ± 0.708	0.008 ± 0.670	−0.164 ± 0.835	<0.001

p, probability.

Data are presented as mean ± standard deviation.

### Cumulative incidences of FLD from normal liver or from borderline FLD over five years

Cumulative incidence of FLD from normal liver or from borderline FLD over five years were 0.65% (52/8,025) and 12.9% (244/1,888), respectively.

### Risk factors for developing FLD

We checked the risk factors for developing FLD from normal liver or from borderline FLD over 5 years, separately. First, we checked the risk factors for developing FLD from normal liver. In crude analysis, younger age, male gender, higher BMI, smoking habits, drinking habits, dyslipidemia, higher SUA levels, and increased SUA change over 5 years are risk factors for developing FLD from normal liver. After multiple adjustments for age, sex, BMI, smoking and drinking habits, dyslipidemia, SUA levels, and SUA change, the risk factors for developing FLD from normal liver are younger age (odds ratio (OR):0.94 per 1 year increased; 95% confidence interval (CI), 0.90–0.97), higher BMI (OR:1.75 per 1 kg/m^2^ increased; 95% CI, 1.55–2.00), higher SUA levels (OR:1.92 per 1 mg/dL increased; 95% CI, 1.40–2.63), and increased SUA change (OR: 3.73 per 1 mg/dL increased; 95% CI, 2.57–5.42) (Table 2).

**Table 2:** Risk factors for developing fatty liver from normal liver function (FLI <30) over five years.

Normal liver		Crude			Adjusted*		
		OR	95% CI	P	OR	95% CI	P
Age	per 1 year increased	0.947	0.919–0.975	<0.001	0.936	0.904–0.968	<0.001
Male gender	versus female gender	3.360	1.918–5.886	<0.001	0.592	0.249–1.405	0.23
Body mass index	per 1 kg/m^2^ increased	1.731	1.545–1.939	<0.001	1.750	1.534–1.996	<0.001
Smoking	positive versus negative	2.399	1.390–4.140	0.002	1.564	0.849–2.882	0.15
Drinking habits	positive versus negative	1.944	1.125–3.360	0.017	1.591	0.846–2.991	0.15
Hypertension	positive versus negative	1.497	0.593–3.780	0.39	0.913	0.327–2.549	0.86
Diabetes mellitus	positive versus negative	—	—	—	—	—	—
Dyslipidemia	positive versus negative	1.814	1.022–3.220	0.042	1.791	0.962–3.335	0.066
Chronic kidney disease	positive versus negative	—	—	—	—	—	—
Serum uric acid	per 1 mg/dL increased	1.766	1.448–2.155	<0.001	1.920	1.400–2.634	<0.001
Serum uric acid change	per 1 mg/dL increased	3.413	2.383–4.889	<0.001	3.734	2.574–5.416	<0.001

OR, odds ratio; CI, confidence interval; p, probability.

There is no subject with diabetes mellitus or chronic kidney disease at the baseline who developed fatty liver over 5 years.

*Data adjusted for age, sex, body mass index, smoking and drinking habits, hypertension, diabetes mellitus, dyslipidemia, chronic kidney disease, serum uric acid and serum uric acid change over 5 years.

Second, we checked the risk factors for developing FLD from borderline FLD. In crude analysis, younger age, higher BMI, and increased SUA change are risk factors for developing FLD from borderline FLD. After multiple adjustments for age and BMI, the risk factors for developing FLD from borderline FLD are younger age (OR:0.95 per 1 year increased; 95% CI, 0.94–0.97), higher BMI (OR:1.15 per 1 kg/m^2^ increased; 95% CI, 1.08–1.23), smoking (OR: 1.37; 95% CI, 1.01–1.87), higher SUA levels (OR: 1.17 per 1 mg/dL increased; 95% CI, 1.02–1.35), and increased SUA change (OR: 1.76 per 1 mg/dL increased; 95% CI, 1.47–2.11) (Table 3).

**Table 3:** Risk factors for developing fatty liver from borderline fatty liver (30 < FLI < 60) over five years.

Borderline fatty liver		Crude			Adjusted*		
		OR	95% CI	P	OR	95% CI	P
Age	per 1 year increased	0.950	0.937–0.963	<0.001	0.956	0.941–0.971	<0.001
Male gender	versus female gender	0.984	0.702–1.381	0.93	0.656	0.420–1.025	0.064
Body mass index	per 1 kg/m^2^ increased	1.188	1.112–1.268	<0.001	1.178	1.096–1.265	<0.001
Smoking	positive versus negative	1.196	0.911–1.570	0.20	1.369	1.005–1.866	0.047
Drinking habits	positive versus negative	1.202	0.915–1.578	0.19	1.305	0.953–1.786	0.097
Hypertension	positive versus negative	0.981	0.691–1.391	0.98	1.152	0.791–1.677	0.46
Diabetes mellitus	positive versus negative	0.373	0.135–1.032	0.057	0.502	0.174–1.447	0.20
Dyslipidemia	positive versus negative	0.843	0.640–1.110	0.22	0.986	0.734–1.323	0.92
Chronic kidney disease	positive versus negative	0.480	0.172–1335	0.16	0.970	0.333–2.821	0.95
Serum uric acid	per 1 mg/dL increased	1.056	0.948–1.177	0.32	1.173	1.023–1.346	0.022
Serum uric acid change	per 1 mg/dL increased	1.658	1.398–1.965	<0.001	1.762	1.468–2.114	<0.001

OR, odds ratio; CI, confidence interval; p, probability.

There is no subject with diabetes mellitus or chronic kidney disease at the baseline who developed fatty liver over 5 years.

*Data adjusted for age, sex, body mass index, smoking and drinking habits, hypertension, diabetes mellitus, dyslipidemia, chronic kidney disease, serum uric acid and serum uric acid change over 5 years.

### Correlation between SUA levels and fatty liver index (FLI)

**Figure 1:**
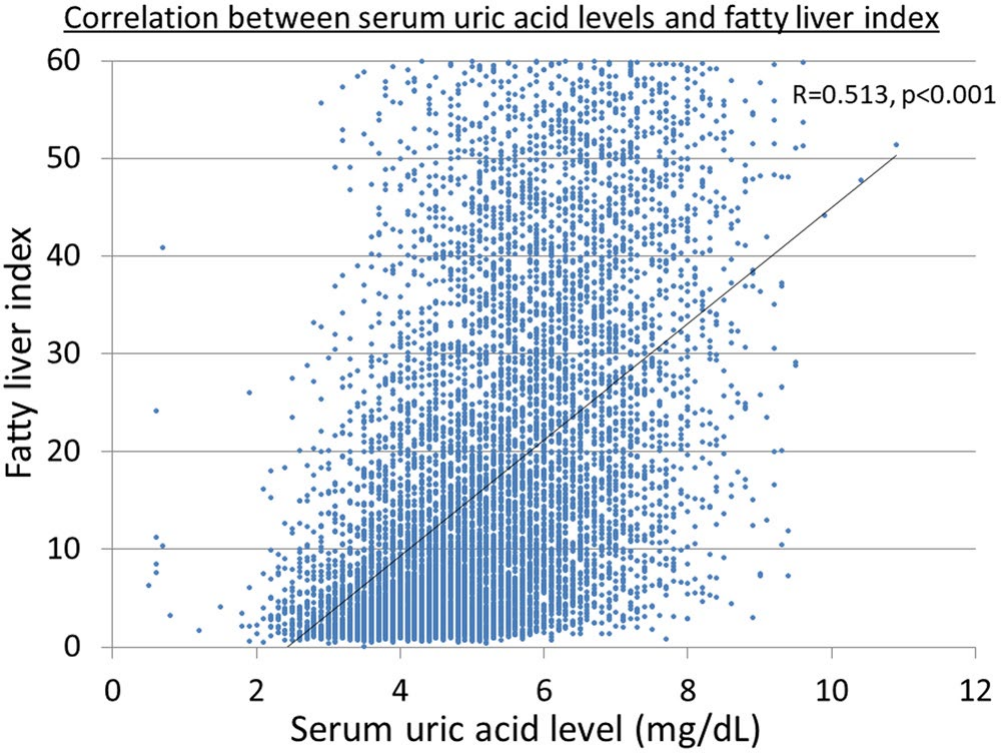
Correlation between serum uric acid levels and fatty liver index. There is significantly positive correlation between serum uric acid levels and fatty liver index by Pearson’s correlation coefficient (R = 0.513, p < 0.001).

**Figure 2:**
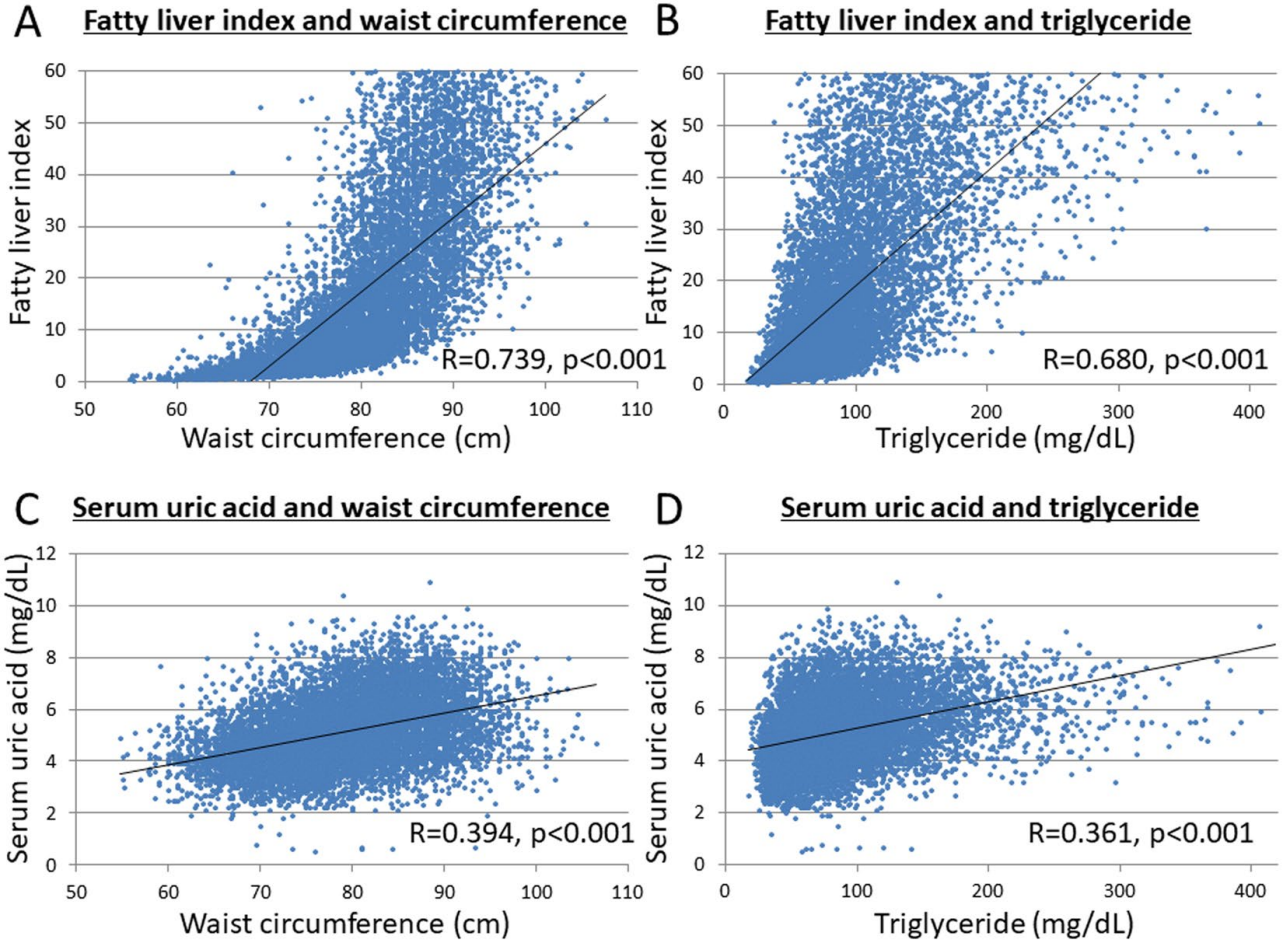
Correlation between fatty liver index, serum uric acid levels, waist circumference, and triglyceride. (**A**) Correlation between fatty liver index and waist circumference There is significantly positive correlation (R = 0.739, *p < 0.001). (**B**) Correlation between fatty liver index and triglyceride There is significantly positive correlation (R = 0.680, *p < 0.001). (**C**) Correlation between serum uric acid levels and waist circumference There is significantly positive correlation (R = 0.394, *p < 0.001). (**D**) Correlation between serum uric acid levels and triglyceride There is significantly positive correlation (R = 0.361, **p < 0.001). All the analyses were conducted by Pearson’s correlation coefficient (R = 0.513, p < 0.001).

We checked the correlation between SUA levels and FLI using all the study subjects’ data (9,914 subjects) in 2004. There is significantly positive correlation between SUA levels and FLI by Pearson’s correlation coefficient (R = 0.513, p < 0.001) (Fig. 1). However, FLI is also influenced by other components of the metabolic syndrome, like waist circumference and triglyceride. We conducted additional analyses of the correlation between FLI and waist circumference (Fig. 2A), between FLI and triglyceride (Fig. 2B), between SUA and waist circumference (Fig. 2C), and between SUA and TG (Fig. 2D). The results showed FLI was more correlated to abdominal circumference (R = 0.739, p < 0.001) or triglyceride (R = 0.680, p < 0.001) than SUA (R = 0.513, p < 0.001). However, the correlations between SUA and waist circumference (R = 0.394, p < 0.001) or between SUA and TG (R = 0.361, p < 0.001) were not as strong (R < 0.4).

## Discussion

The primary goal of our study was to identify risk factors for developing FLD in a general healthy Japanese population. In the subjects both with normal liver and borderline FLD at the baseline, not only higher SUA levels but also increased SUA change over 5 years became independent risk factors for developing FLD over 5 years, as well as younger age and higher BMI. Moreover, increased SUA change was the highest OR for developing FLD after multiple adjustments. Our study also showed significantly positive correlation between baseline SUA levels and FLI.

The key finding in our study is that, not only does baseline SUA predict the development of FLD in a healthy population independent of several other traditional risk factors, but increased SUA change over 5 years also becomes a strong risk for developing FLD. There are many studies that clarified the association between SUA and FLD, but this is the first study that assessed SUA change over 5years as a risk factor for developing FLD. While several studies have found elevated SUA associated with presence or severity of FLD^11,15–22^, others have not including in an adolescent and adult female population^23,24^. Some have contended that SUA elevations seen in metabolic syndrome are a result of the pathology rather than a cause of the pathology^25–28^. Also, some contend that hyperinsulinemia decreases renal uric acid excretion and that uric acid is an innocent bystander^26^. Yet our findings are consistent with other longitudinal cohort studies that did find SUA levels were a predictor of FLD development in Korean and Chinese adult populations^12–14^. In addition, SUA is associated with more advanced FLD in adolescent and adult populations^17,18,29^, and elevated baseline SUA predicted cirrhosis related hospitalization or death even after adjustment for causes or risk factors of chronic liver disease in 5,518 patients during a 12.9 year median follow up^15^.

There is also data that supports a role in lower SUA levels leading to improvement in FLD. Animal FLD models have shown use of allopurinol or febuxostat improves markers of inflammation as well as in a human liver line (HepG2)^30,31^. In addition, a small study in humans found allopurinol improved levels of cytokeratin 18 (marker for hepatocyte apoptosis), AST, ALT, total cholesterol, and Triglycerides^32^. Finally, a longitudinal cohort found lower SUA levels at baseline predicted resolution of FLD^33^. A recent study by Cicero, A. F. G. *et al*. showed that SUA and FLI were also related to pulse wave velocity^34^. The study suggested that arterial stiffness might be associated with not only high SUA levels, but also FLD.

An interesting finding is that for patients with borderline FLD, SUA is smaller risk than the normal liver population. As noted in Table 1, borderline FLD patients are older, weigh more with greater waist circumference, and have more features of metabolic syndrome (dyslipidemia, higher blood pressure, and presence of diabetes) compared to the normal liver group. SUA has been strongly linked to playing a role in the development of metabolic syndrome^35^ and therefore it is possible that SUA leads to FLD through indirect mechanism such as insulin resistance than direct effects on liver pathology. As of note, our normal liver population had few features of metabolic syndrome, and even after adjustment for traditional risk factors, found SUA to strongly associate with the development of FLD. It has been shown that uric acid directly drives oxidative stress in HepG2 cells and this further activates endoplasmic reticulum (ER) stress along with activating genes involved in lipogenesis^36,37^. Another study found an association of SUA with lean FLD^38^. Thus, SUA level at baseline and increases in SUA highlight a population at risk for FLD particularly in a relative healthy appearing population compared to borderline FLD group. Evidence supports uric acid’s role in FLD, both via direct means on ER stress and lipogenesis as well as indirect means such as weight gain, and insulin resistance^39^, which drive free fatty acid shunting to the liver and de-novo lipogenesis^40^. Our study does not clarify, which mechanism could potentially be playing the more prominent role.

We found that a younger age at baseline predicted the development of FLD. Dietary habits in younger patients compared to older patients are likely more of a Western diet than traditional Japanese food^41,42^. In addition, it may be that older patients who already have FLD were excluded since they had FLI ≥ 60 and those (of the older patients) who had a FLI <60 are not as likely to develop FLD if they have factors (ie healthier diet) placing them at less risk for development of FLD. In addition, for both groups, BMI was found to be risk factor for development of FLD.

Our study has several limitations. First, this study is a retrospective single center study, which may have introduced selection bias. However, single center studies have some advantages of the similarity of methodology. Second, we did not exclude for elevated bilirubin and other liver enzymes at the baseline, which might suggest another liver disorder and could affect SUA levels as there is association between jaundice and hypouricemia^43^. However, this study population was done in a generally healthy population. Data from Table 1 shows average bilirubin and other liver enzymes were normal for this population. In our multivariable analysis, we used a category of dyslipidemia which included triglyceride. However, we did not include abdominal circumference because we included BMI. BMI is strongly correlated with abdominal circumference, and we used only BMI in the multiple adjustment models to exclude confounding bias. Third, the FLI is designed to identify those with low likelihood of FLD. Use of FLI is a limitation since it is an indirect measurement of FLD. It is possible some patients in the borderline FLD already had FLD and maybe possible that even some with healthy liver may have FLD while some with an FLI ≥ 60 do not, though, as stated before, a FLI < 30 strongly rules out likelihood of FLD disease, while an FLI ≥ 60 strongly indicates likely FLD. FLI has been validated in Asian populations (Korean cohort)^44^ and recent research has shown FLI to accurately predict steatosis when compared to MR Spectroscopy (MRS)^45^. Fourth, our study did not separate out alcoholic FLD (AFLD) from nonalcoholic FLD (NAFLD). We adjusted for drinking habits though, and found both baseline SUA and changes in SUA still to be risk factors for the development of FLD suggesting SUA plays a role in NAFLD, though it could likely play a role in AFLD as well. Of note, drinking habits was not associated with risk of FLD development. In addition, there is likely coexistence of AFLD and NAFLD in the population^46^. Finally, this is an observation study, and it cannot show the causal relationships between SUA and FLD. Intervention studies are needed to clarify whether the treatments for hyperuricemia in subjects with normal liver are useful to prevent the development of FLD.

In conclusion, not only higher baseline SUA, but also increased SUA change over 5 years become independent risks for developing FLD both from normal liver and borderline liver, as well as younger age and higher BMI. Therefore, in a relative healthy population we should pay attention to both baseline SUA and SUA change as risk factors for developing FLD. Further research and especially clinical trials are needed to evaluate whether SUA lowering can impede the development of FLD.

## Methods

### Study design

This is a retrospective, single-center, cohort study in Japan to clarify the risk factors for developing FLD. The database was from the Center for Preventive Medicine, St. Luke’s International Hospital, Tokyo, Japan. The study analyzed 13,201 Japanese subjects who underwent annual medical examinations at the center in 2004 and were reevaluated five years later. We used FLI to assess FLD^47^. This is a well established algorithm based upon BMI, waist circumference, triglycerides, and gamma-glutamyl-transferase (GGT) that is shown to have an accuracy of 84% with a cutoff value of <30 to rule out hepatic steatosis (sensitivity of 87% and a negative likelihood ratio of 0.2) and FLI ≥ 60 to rule in (Specificity 86%, positive likelihood ratio 4.3). We included the subjects between the ages 20 and 75 years old at the baseline (2004) given this was the age cutoffs in the validation study^47^. We excluded the subjects with hepatitis B (HBs antigen positive) and C (HCV antibody positive) virus infection and subjects with FLD (FLI ≥ 60) at the baseline. We also excluded the subjects on medication for hypertension, dyslipidemia, diabetes mellitus, and hyperuricemia and/or gout. When the subjects had more than one annual examination, we only used the first examination of that year to avoid counting the individual twice. Every subject had the same work-up, including medical history, routine physical examination, and blood tests collected in this database and detailed in previous publications^48–51^. We evaluated the cumulative incidence of FLD from normal liver (FLI < 30) and borderline FLD (30 ≤ FLI < 60) at baseline. Moreover, we evaluated risk factors for developing FLD both from normal liver and from borderline FLD over five years and calculated ORs. Finally we prefer to use the term FLD over NAFLD even though we adjusted for drinking habits given total intake was not assessed nor were other rare causes of FLD such as celiac or certain medications eliminated from analysis^52^.

### Fatty liver index (FLI)

FLI is calculated by this formula^47^.

$$\begin{array}{c}{\rm{FLI}}=({{\rm{e}}}^{0.953\ast {\rm{loge}}({\rm{triglycerides}})+0.139\ast {\rm{BMI}}+0.718\ast {\rm{loge}}({\rm{GGT}})+0.053\ast {\rm{waist}}{\rm{circumference}}-15.745})\\ \,(1+{{\rm{e}}}^{0.953\ast {\rm{loge}}({\rm{triglycerides}})+0.139\ast {\rm{BMI}}+0.718\ast {\rm{loge}}({\rm{GGT}})+0.053\ast {\rm{waist}}{\rm{circumference}}-15.745})\ast 100.\end{array}$$

GGT, Gamma-glutamyl transferase

### Study subjects

**Figure 3:**
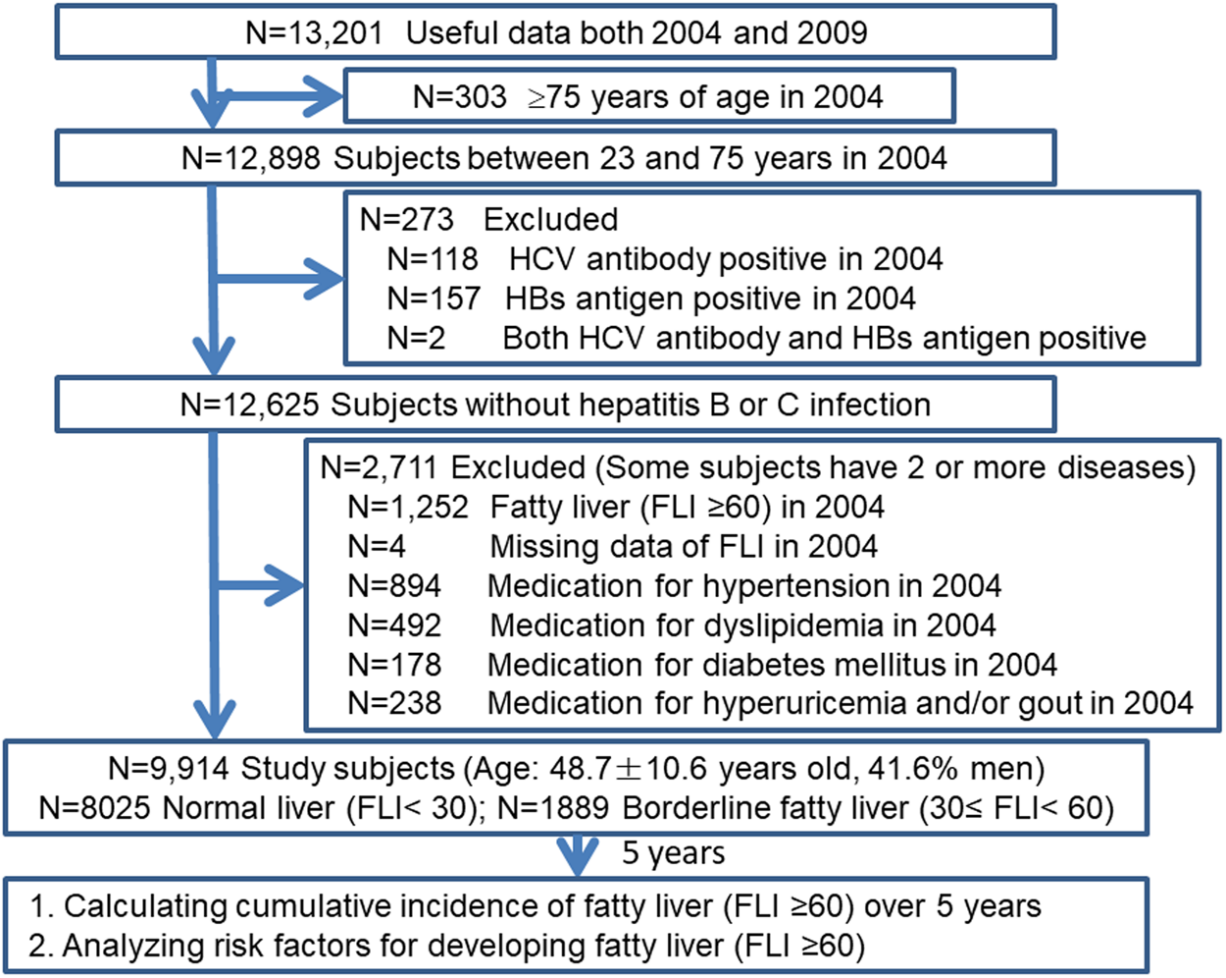
Flow diagram of study enrollment N: number of subjects; FLI, fatty liver index.

Out of the 13,201 subjects, no subject was less than 20 years old (23 years old is the youngest in this study) and 303 subjects were 75 years old and above in 2004. Of 12,898 subjects, 118 subjects were HCV positive and 157 subjects were HBs antigen positive (2 subjects had both). Of 12,625 subjects without hepatitis virus infection, we excluded 1,252subjects with FLD, 4 subjects with missing data of FLI, 894 subjects on medication for hypertension, 492 subjects on medication for dyslipidemia, 178 subjects on medication for diabetes mellitus, and 238 subjects on medication for hyperuricemia and/or gout. Finally, we analyzed 9,914 subjects (age: 48.7 ± 10.6 years old, 41.6% men) (Fig. 3).

### Patient involvement

No patients were involved in setting the research question or outcome measures, nor were they involved in the design and implementation of the study. There are no plans to involve patients in dissemination.

### Definition of hypertension, diabetes mellitus, dyslipidemia, and chronic kidney disease

Hypertension is defined as a condition when subjects are on current antihypertensive medication and/or systolic blood pressure of greater than or equal to 140 mmHg and/or diastolic blood pressure of greater than or equal to 90 mmHg^53,54^ blood pressure readings were obtained using an automatic brachial sphygmomanometer (OMRON Corporation, Kyoto, Japan), which were upper arm blood pressure measurements that had passed validation. Two blood pressure examinations were taken after the participants were seated and rested quietly for more than five minutes with their feet on the ground and their back supported. The mean systolic and diastolic blood pressure of each of the subjects were calculated from the recorded measurements. Diabetes mellitus is defined as current diabetes mellitus on medication use and/or HbA1c (National Glycohemoglobin Standardization Program) greater than or equal to 6.5%, according to International Expert Committee^55^. Dyslipidemia is defined as current medication use for dyslipidemia and/or low-density lipoprotein cholesterol greater than or equal to 140 mg/dl, high-density lipoprotein cholesterol less than 40 mg/dL, and/or triglycerides greater than or equal to 150 mg/dL, according to Japan Atherosclerosis Society guidelines^56^. Chronic kidney disease is defined as estimated glomerular filtration rate (eGFR) less than 60 mL/min/1.73 m^2^. We calculated eGFR using the Japanese GFR equation: eGFR (mL/min/1.73 m^2^) = 194 × serum creatinine^−1.094^ × age^−0.287^ (×0.739 if woman)^57^. Smoking habits included past smoking and current smoking. Drinking habits were defined as drinking alcohol every day or not.

### Statistical analysis

Statistically significance was set at a probability (p) value < 0.05 (two sided). Data are expressed as mean ± standard derivation or as percent frequency unless otherwise specified. Comparisons between two groups were performed with student *t*-tests for normally distributed variables, and χ^2^ analyses for categorical data. The risk factors for developing FLD from normal liver or borderline FLD in the period of over five years were evaluated both by non-adjusted (crude) models and by multivariable logistic regression models. We checked risk factors for developing FLD with age, sex, body mass index (BMI), smoking and drinking habits, hypertension, diabetes mellitus, dyslipidemia, chronic kidney disease, baseline SUA levels, and SUA change over 5 years (calculated by SUA in 2009 – SUA in 2004) by crude models and multivariable adjusted models, and odds ratios (ORs) were analyzed in each group. We also checked the correlation between SUA levels and FLI by Pearson’s correlation coefficient. All the statistical analyses were performed using the SPSS Statistics software (IBM SPSS Statistics version 22 for Windows; IBM, New York, USA).

### Ethical considerations

We adhered to the principles of the Declaration of Helsinki. All data were collected and compiled in a protected computer database. Individual data were anonymous without identifiable personal information. Informed consent was obtained from all subjects by a comprehensive agreement method provided by St. Luke’s International Hospital. St. Luke’s International Hospital Ethics Committee approved the protocol for this study.
